# Attitudes and health behaviors of middle-aged and older adults with elevated tumor markers in China

**DOI:** 10.3389/fpsyg.2024.1265648

**Published:** 2024-02-06

**Authors:** Renke Yu, Zhijie Xu, Yiting Lu, Yue Zhu, Liying Chen

**Affiliations:** ^1^Department of General Practice, Sir Run Run Shaw Hospital, Zhejiang University School of Medicine, Hangzhou, China; ^2^Department of General Practice, The Second Affiliated Hospital, Zhejiang University School of Medicine, Hangzhou, China; ^3^Department of General Practice, Zhongdai Community Healthcare Center, Huzhou, China

**Keywords:** tumor markers test, attitudes, behaviors, cancer screening, lifestyle, information seeking

## Abstract

**Objectives:**

To understand the attitudes and health behaviors of middle-aged and older adults in China after receiving elevated results of tumor markers (TMs) test in the annual health examinations (AHEs) and explore the influencing factors.

**Methods:**

A three-section online questionnaire survey was conducted from March 1 to April 30, 2020 in Hangzhou, China, to people who were aged 45 and older and had at least one elevated result of TMs test. Clinical information was collected from the online survey and medical records. Descriptive statistics were carried out followed by regression analyses.

**Results:**

Of 380 participants, 76.1% were unwilling to quit the TMs test in AHEs, whereas 75.3% would take the doctor’s advice and quit unnecessary TMs test; 67.4% felt stressed about their TMs. Among participants with elevated TMs, 76.8% changed lifestyle to keep healthy, 74.2% sought health information, 58.9% requested a TMs retest, and 50.3% did further tests to confirm a diagnosis. Family history of cancer was associated with lifestyle changing; education level, area of residence and health insurance were associated with health information seeking; comorbidity were associated with retests and sequential confirming tests.

**Conclusion:**

The application of the TMs test in AHEs among Chinese people may lead to positive and negative behavioral consequences and psychological distress. Doctors have a significant impact on patients’ health behaviors. Accurate indications and adequate communication with patients before and after the TMs test are in great need.

## Introduction

Annual health examinations (AHEs) are becoming increasingly common in China. A total of 373 million people underwent the AHEs in 2014; this number has increased by 62.17% from 230 million in 2009 in China (Zhiyan Consulting Group, 2016). Overall, 77.30% AHEs were conducted in comprehensive hospitals, and the rest in community health centers or private commercial medical centers (Zhiyan Consulting Group, 2016). Considering the popularization and promotion of cancer screening in the Chinese population, cancer screening is a major part of AHEs and tumor markers (TMs) test is a common item (Dong et al., 2008). A recent study indicated that TMs test requests were 13 million per year in Italy, which was considerably higher than expected (Gion et al., 2016). Although there is no authoritative accurate statistical data of China, the TMs test requests per year may be enormous, considering the size of population in China.

The value of TMs test in AHE remains controversial. On the one hand, TMs test is a convenient approach for cancer screening. Tumor cells generate some molecules into circulation and higher levels can be detected so that these molecules are given the name of “TM.” Cause TMs can be indicative of corresponding tumors (Modest and Heinemann, 2015) and need only simple blood-tests, TMs are widely applied for early-diagnosis, staging, prognosis, detecting recurrence, and monitoring therapy for cancer (Sturgeon, 2002). For example, measuring prostate-specific antigen (PSA) as a screening parameter in AHEs was useful to detect prostate cancer early with a detection rate of 2.1% in males older than 50 years (Uchida et al., 2000). A previous study demonstrated that a multi-analyte biomarker panel was more sensitive in the detection of cancers in AHEs than a single marker, especially for hepatocellular carcinoma and prostate cancer (Wen et al., 2015). During follow-ups for people with elevated TMs after AHEs, more causative diseases might be found, such as several types of cancers, chronic diseases, and infections (Lee et al., 2019).

However, TMs tests are not recommended in the screening of a healthy population because of their unsatisfactory sensitivity and specificity (Sturgeon, 2002; Modest and Heinemann, 2015). According to a large health checkup cohort study, the percentage of general population with elevated carbohydrate antigen 19–9 (CA 19–9) was approximately 1% and only 0.04% were diagnosed with cancer, implying that the application of CA 19–9 test for cancer screening in the healthy population was inappropriate (Wang et al., 2016; Lee et al., 2019). Similarly, the colorectal cancer (CRC) detection rate in CRC screening programs was only 0.25% in Taiwan and 0.18% in Canada (Navarro et al., 2017). Application of TMs test to general population without high cancer-risk may cause the waste of medical resources.For example, the suspicious results revealed by TMs test need sequential tests and treatment (e.g., repeat testing, imaging, endoscopic procedures, and surgeries) which may account for the major proportion of health care expenditure (Zhi et al., 2013), increasing the medical burden.

The result of TMs may be more meaningful in the aspect of prognosis of malignant tumors. European Group on Tumor Markers recommends the use of carcinoembryonic antigen (CEA) and carbohydrate antigen 15–3 (CA15-3) levels for prognosis assessment of breast cancer (Molina et al., 2005). A previous study showed esophageal neuroendocrine carcinoma patients with elevated neurone specific enolase (NSE) had a significant worse prognosis than the ones with a normal NSE value (Sohda et al., 2021). Pang H. et al. found CA199 together with body mass index and the count of lymphocytes showed an effective predictability of overall survival in gastric cancer patients (Pang et al., 2021). Therefore the TMs may play a more important role in the prognosis prediction of tumors (Perelli et al., 2023).

The TMs test can be mandated by doctors or requested by patients as an additional service. People’s attitudes and knowledge of TMs test can influence the health-related medical decision making in some degree. People’s reactions after getting the TMs results are also very important, including cancer worry, sequential test requests, lifestyle changing, information seeking, psychological problems and so on. However, there are few studies focus on this, especially for those who want to do cancer screening and get elevated TMs. This study aims to investigate the attitude of people requesting TMs test in AHEs, and the behavior of those with elevated results as well as the association factors.

## Methods

### Design and setting

We conducted an online survey from March 1 to April 30, 2020, across Wenjuanxing, an online questionnaire platform in China. The questionnaire was adapted from the Medication Attitudes Module and its reliability and validity were verified (Reeve et al., 2018). The questionnaire consisted of three sections, including items on participants’ sociodemographics, attitudes, and behaviors related to taking the TMs test. The first section on sociodemographic information included age, the region of residence, gender, educational level, and health insurance. In the second section, eight statements assessing the attitudes toward the TMs test were answered with “strongly agree,” “agree,” “disagree,” and “strongly disagree.” The participants’ attitudes toward the TMs test were presented as proportions (%). We carried out two statements of eight statements into regression analyses: “I would be willing to quit the unnecessary TMs test in AHE” and “I do not want to quit the TMs test in AHE,” which had the best representativeness. The third section included six questions about the behaviors after receiving elevated results on the TMs test. These behaviors included changing lifestyle, seeking health information about tumor markers, and requesting TMs retest or more tests for confirming a diagnosis. The questionnaire took five to 10 min to complete.

The study was approved by the Sir Run Run Shaw Hospital Ethics Committee and was conducted in accordance with the Declaration of Helsinki (World Medical Association, 2013). This survey was conveyed through the Internet as e-questionnaire by massages and follow-up calls were made to individuals who had not responded to the questionnaire to seek their agreement to participate in the study. All participants were provided online informed consent before the e-questionnaire. Only if the participants agreed with the online informed consent that could the following questionnaire be done. Participants did not receive any compensation for their participation.

### Participants

We included people aged 45 years and older who underwent the AHE in Sir Run Run Shaw Hospital from January 1 to December 31 2018, and had at least one elevated tumor marker. We focused on four TMs included in all AHEs: (a) CEA (normal range ≤ 5.00 ng/mL); (b) alpha-fetoprotein (AFP, normal range ≤ 8.78 ng/mL); (c) CA19-9 (normal range < 37.00 U/mL); (d) NSE (normal range < 16.30 ng/mL). The AFP and CEA were measured using kits from Abbott Diagnostics, while CA19-9 and NSE were measured using kits from Roche Diagnostics. We excluded individuals who were previously diagnosed with a malignant tumor or a known mental illness. We randomly selected a total of 778 people who met the inclusion criteria to participate in this study and sent the link to the questionnaire via a text message. And 380 of them responded, aged range from 45 to 70 years old (mean age 53.92 ± 6.24 years old), and 246 (64.7%) were men.

**Patient and Public Involvement statement**: The patients did not participate in the design of, recruitment to and conduct of the study. We invited patients to participate in our study and it’s up to them to decide whether to participate or not. And we gave 5 RMB yuan to each participant as thanks. The survey was conducted thorough an online questionnaire and the e-link of the questionnaire was conveyed by telephone messages.

### Data collection

Questionnaire data were collected digitally and then exported into a spread sheet. The clinical information was collected from the electronic medical records (EMR) of Sir Run Run Shaw hospital, and included symptoms, family history of cancer, and comorbidities.

### Statistical analysis

Statistical analyses were performed using Stata (StataCrop LLC) version 15.0 to present the distribution of sociodemographic and clinical characteristics, as well as behaviors after receiving elevated results in the TMs test. The results of behaviors were reported in frequency, percentage, and 95% confidence interval (95% CI). We classified the responses into two levels of agreement (“strongly agree” or “agree”) and disagree (“disagree” and “strongly disagree”). The content of the survey was shown in the Supplementary material 1. Multivariable logistic regression was used to determine the association among characteristics, attitudes, and behaviors, presented in odds ratios (OR) and 95% confidence intervals (CI). Some OR has been adjusted by related confounders and represented by OR*. A 2-sided *p* < 0.05 was considered statistically significant.

## Results

### Participants characteristics

For the 380 respondents (response rate: 48.8%), there was no incomplete questionnaire. Among them, 318 (83.7%) were 45–59 years old and 62 (16.3%) were older than 60 years old. A total of 208 respondents (54.7%) had a college degree or higher and 226 (70.0%) were urban residents. Most participants, 323 (85.1%), had health insurance; 235 (61.8%) reported at least one comorbidity, such as hypertension (22.4%), hyperlipidemia (18.7%), fatty liver (11.3%), diabetes (7.4%) (Table 1).

**Table 1:** Sociodemographic and clinical characteristics of participants.

Variable	Frequency Total (*n* = 1,180)	Percentage (%;95%CI)
Age (years)		
45–59	318	(83.7;79.6–87.1)
≥60	62	(16.3;12.9–20.4)
Gender		
Male	246	(64.7;59.8–69.4)
Female	134	(35.3;30.6–40.2)
Education		
High School and below	172	(45.3;40.3–50.3)
College degree and higher	208	(54.7;49.8–59.7)
Area of residence		
Urban	226	(70.0;65.2–74.4)
Rural	114	(30.0;25.6–34.8)
Health insurance		
Yes	323	(85.1;81.0–88.3)
No	57	(14.9;11.7–19.0)
Symptoms		
Yes	164	(43.1;38.2–48.2)
No	216	(56.9;51.8–61.8)
Family history of cancer		
Yes	100	(26.4;22.1–31.0)
No	280	(73.6;69.0–77.9)
Comorbidities		
None	145	(38.2;33.4–43.2)
1	116	(30.5;0.26–35.4)
≥2	119	(31.3;26.8–36.2)

There were 55 participants with elevated CEA, 47 of AFP, 46 of CA19-9, and 232 of NSE. Among them, 24 participants had two elevated TMs. The average level of CEA was (6.54 ± 1.50) ng/ml, AFP (12.04 ± 4.04) ng/ml, CA19-9 (57.47 ± 38.25) U/ml, NSE (19.84 ± 4.28) ng/ml.

### Attitudes toward the TMs test

**Figure 1:**
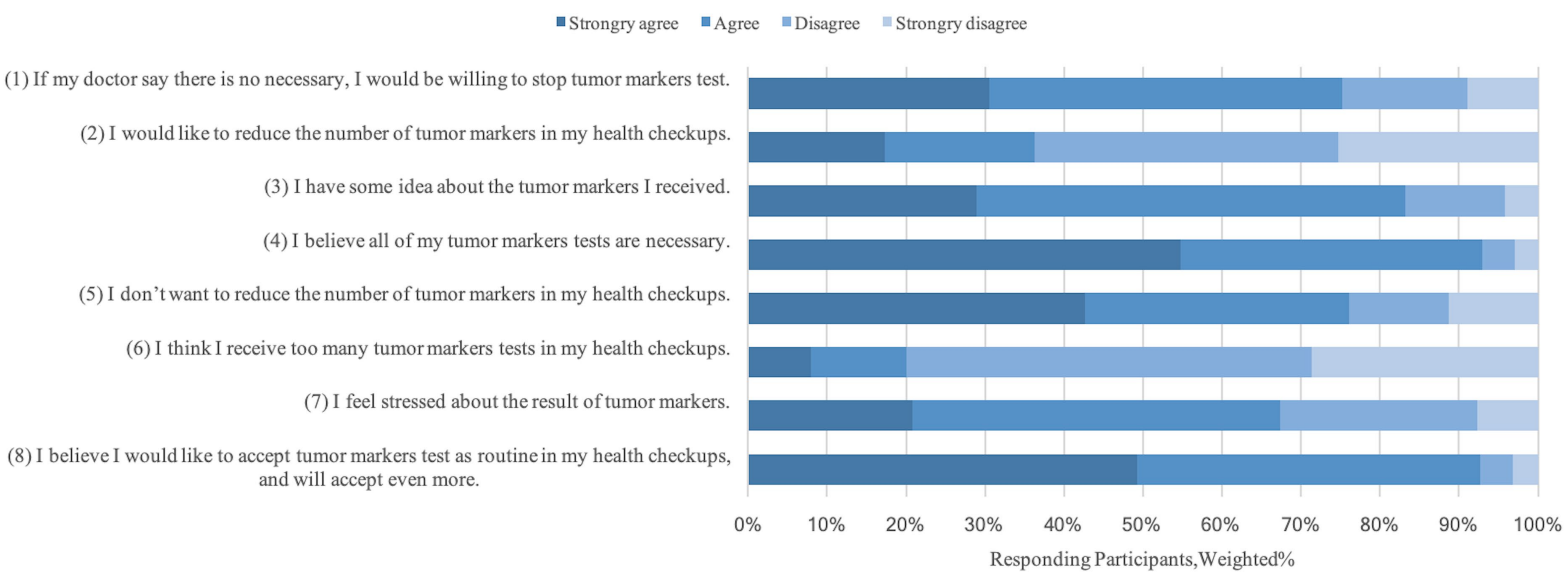
Patients’ attitudes to the test of tumor markers.

A total of 289 (76.1%) participants reported that they would not like to quit the TMs test in AHE, and the majority, 92.6%, reported that they would be willing to receive more types of TMs test. However, 286 (75.3%) reported that they would take their doctor’s advice and quit the unnecessary TMs test (Figure 1). More than two-thirds, 67.4%, reported feeling stressed about their elevated TMs.

### Behaviors after getting elevated TMs

After the participants were found to have elevated TMs, approximately 76.8%, tried changing their lifestyle to keep healthy, such as choosing a healthy diet, smoking cessation, reducing alcohol consumption, and engaging in more physical activities (Li et al., 2018). A total of 282 (74.2%) participants sought health information about TMs, and their approaches ranged from consulting doctors in general hospitals (49.6%) to searching the Internet (38.7%) and consulting family doctors or friends (11.7%). More than half (58.9%) requested at least one TMs retest and 50.3% of participants underwent more tests to confirm a diagnosis (e.g., enhanced computed tomography (CT) scan, endoscopic procedures, biopsy).

### Factors associated with behaviors after getting elevated TMs

Multivariable logistic regression was done to figure out the association among characteristics, attitudes, and behaviors, showed by OR and 95%CI. These confounders should be taken into account, like age, gender, education, area of residence, health insurance, symptoms, family history of cancer and comorbidities. For “Getting more tests to confirm a diagnosis,” there was no association between these confounders with “Getting more tests to confirm a diagnosis” respectively, so we did not adjust for these OR. For “Changing lifestyles,” there was an association between family history of cancer and “Changing lifestyles.” Then, we adjusted this one confounder in the regression analysis of the association between attitudes and behaviors, and got adjusted OR*s. For “Seeking health information about tumor markers,” education, area of residence, health insurance was associated with “Seeking health information about tumor markers.” So, we adjusted these three confounders in the regression analysis of the association between attitudes and behaviors, and got adjusted OR*s. Similarly, for “Getting a tumor markers retest,” comorbidities were associated with “Getting a tumor markers retest,” so we adjusted this one confounder in the regression analysis of the association between attitudes and behaviors, and got adjusted OR*s. Participants’ willingness to change lifestyle was significantly associated with their family history of cancer (Table 2). People with a family history of cancer were more likely to change their lifestyles (OR 1.21, 95%CI 1.09–1.34). Education level, area of residence, and health insurance were associated with seeking health information. Participants with a college degree or higher (OR 1.21, 95%CI 1.06–1.36), and owning health insurance (OR 1.29, 95%CI 1.03–1.61) were more likely to seek health information. Participants with one comorbidity (OR 1.27, 95%CI 1.03–1.57) and more than two comorbidities (OR 1.29, 95%CI 1.04–1.58) were more likely to receive TMs retests, whereas those refusing to quit the TMs test in AHE were less likely to receive TMs retests (OR* 0.77, 95% CI0.65–0.91) and sequential confirming tests (OR 0.72, 95%CI 0.59–0.87).

**Table 2:** Multiple logistic regression of participants’ behaviors after getting elevated result of tumor markers test.

	Changing lifestyles	Seeking health information about tumor markers	Getting a tumor markers retest	Getting more tests to confirm a diagnosis
	OR (95%CI)	OR (95%CI)	OR (95%CI)	OR (95%CI)
Age (years)				
45–59	1.00	1.00	1.00	1.00
≥60	1.01 (0.87–1.17)	0.95 (0.80–1.13)	1.12 (0.91–1.37)	0.92 (0.69–1.23)
Gender				
Male	0.90 (0.81–1.00)	0.93 (0.83–1.05)	0.87 (0.74–1.03)	0.85 (0.70–1.04)
Femle	1.00	1.00	1.00	1.00
Education				
High School and below	1.00	1.00	1.00	1.00
College degree and higher	0.98 (0.87–1.09)	1.20 (1.06–1.36)	0.99 (0.84–1.17)	0.96 (0.78–1.17)
Area of residence				
Urban	1.00	1.00	1.00	1.00
Rural	0.99 (0.88–1.12)	0.80 (0.69–0.93)	0.95 (0.79–1.15)	1.05 (0.85–1.30)
Health insurance				
Yes	1.11 (0.93–1.33)	1.29 (1.03–1.61)	1.19 (0.91–1.55)	0.98 (0.74–1.29)
No	1.00	1.00	1.00	1.00
Symptoms				
Yes	1.01 (0.91–1.13)	0.95 (0.84–1.07)	0.99 (0.83–1.17)	0.96 (0.78–1.17)
No	1.00	1.00	1.00	1.00
Family history of cancer				
Yes	1.21 (1.09–1.34)	1.00 (0.87–1.14)	0.98 (0.81–1.19)	0.95 (0.75–1.20)
No	1.00	1.00	1.00	1.00
Comorbidities				
None	1.00	1.00	1.00	1.00
1	1.04 (0.91–1.19)	0.98 (0.85–1.13)	1.27 (1.03–1.57)	1.11 (0.87–1.41)
≥2	1.03 (0.90–1.18)	0.92 (0.80–1.07)	1.29 (1.04–1.58)	1.06 (0.83–1.36)
I would be willing to quit the unnecessary tumor markers test in AHE				
Agree	1.00* (0.88–1.14)	0.94* (0.83–1.07)	0.88* (0.73–1.05)	0.90 (0.72–1.12)
Disagree	1.00	1.00	1.00	1.00
I do not want to quit tumor markers test in AHE				
Agree	0.96* (0.85–1.09)	0.95* (0.84–1.09)	0.77* (0.65–0.91)	0.72 (0.59–0.87)
Disagree	1.00	1.00	1.00	1.00
I feel stressed about the result of tumor markers.				
Agree	1.10* (0.97–1.25)	1.02* (0.89–1.16)	1.04* (0.86–1.25)	0.98 (0.79–1.21)
Disagree	1.00	1.00	1.00	1.00

OR: odds ratio; CI: confidence interval.

OR*: the OR value with *means adjusted OR.

## Discussion

To our knowledge, this is the first study to investigate the attitudes and health behaviors of people with elevated TMs in AHEs. An online questionnaire survey was conducted to understand people’s attitudes and health behaviors toward the TMs test, and multivariable logistic regression was performed to explore the influencing factors.

This study’s findings showed that the TMs test was widely accepted by participants in AHEs with the aim of cancer screening and most participants had a strong willingness to repeat the TMs test in the next AHE or receive even more types of TM tests. A previous study showed that people had a strong willingness to undergo cancer screening despite inadequate previous awareness of the disadvantages (Goto et al., 2020). A large population-based cohort study indicated that testing in people with low risk in an AHE increases the possibility of subsequent outpatient visits, diagnostic tests, and procedures (Bouck et al., 2020). Most people undergoing an AHE in China are asymptomatic and with low risk of cancer. Therefore, the requests for TMs tests are blinded to some contexts that may potentially lead to unnecessary medical expenditure.

Doctors’ professional advice may change people’s willingness to undergo the TMs test. In our study, a large proportion of participants agreed on quitting the unnecessary TMs test in the future AHE if the doctors advised so. Doctors seemed to play a vital role in the decision of taking TMs test. Consistent with our findings, Goto et al. suggested that receiving cancer screening was associated with medical support from primary care physicians. People with a primary care physician are more likely to adhere to the recommendations for cancer screening (Goto et al., 2018). However, another study found that people would like to undergo subsequent cancer screening even if they were informed of the disadvantages, such as overdiagnosis, false positives, and false negatives (Goto et al., 2020). The contradictory results may somewhat be explained by the way physicians communicate. The communication between physicians and people before the TMs tests may be inadequate. Only a small proportion of people (14%) were informed of the disadvantages of the PSA test and (10%) had discussions before the test with their health care professionals (Leyva et al., 2016). Only 36% women were well informed before breast cancer screening (Charaka et al., 2021). This shows that doctors’ professional explanations and communication have a significant impact on people’s health behaviors in cancer screening, which is not adequate at the moment. As “gate-keepers” of clinical tests and examinations, doctors are expected to follow accurate indications, and more efforts should be made by doctors to communicate effectively with people before the TMs test. Communication with patients is supposed to be an important part of medical education. We advocate that prior communication with the participants will lead to a better outcome. Furthermore, detailed and understood explanations including value and disadvantages before TMs test are indispensable to rational application of TMs test.

The elevated results of the TMs test may have a psychological impact on people. In our study, more than two-thirds of participants felt stressed about their elevated TMs. Similarly, a systematic review demonstrated that a cancer screening program had a psychological effect on participants, especially among those with positive results (Vermeer et al., 2017). The psychological distress may have prolonged effects after the test. For instance, psychological distress remained up to six months after the positive results of the fecal occult blood test in colorectal cancer screening (Vermeer et al., 2020). Furthermore, the sequential tests (e.g., biopsy) can bring additional distress, especially for people who obtain positive findings or without a definitive result (Sharp et al., 2018). Although some studies indicated that the psychological harms of cancer screening were generally slight in severity and short in duration (Chad-Friedman et al., 2017), the lack of uniformity of the evaluation tools to measure the psychological distress may account for these ambivalent consequences (Cossu et al., 2018).

An abnormal result in the TMs test in AHE also might lead to changes in participants’ health behaviors. Approximately three-quarters of the participants in our study reported trying to improve their lifestyles to keep healthy. Arousing people’s attention to health is one of the positive consequences of the wide utilization of the TMs test in AHE. Although mild elevated TMs are far from being a cancer diagnosis, people may try to eat healthily, quit smoking, reduce alcohol consumption, and engage in more physical activities. About three-quarters of the participants sought health information after the tests; most of them turned to doctors in comprehensive hospitals, and some through the Internet. The information-seeking behaviors indicated that patients regarded their physicians as information givers (Adamson, 2018).

Our study showed that many factors were associated with people’s health behaviors about the TMs test. For example, participants with a family history of cancer were more willing to improve their lifestyles after receiving an elevated result of TMs. Previous studies showed that male adults with a family history of cancer were more likely to have better health behaviors compared to females (Hwang et al., 2019). The awareness of the relationship between the lifestyle risk factors and cancer may account for the difference in health behaviors. Some people reported being uncertain about the importance of the lifestyle in reaction to cancer (Anderson et al., 2017). Health education about the high-risk factors of cancer is helpful for people to deal with cancer screening properly.

In this study, participants with higher education levels, urban residence, and health insurance were more inclined to seek health information about cancer. Previous studies provided evidence that people with higher education levels were more likely to seek cancer information (Kaphingst et al., 2009). People with higher education levels are likely to have a better basis of knowledge and health literacy; thus, they have the ability and sensibility to seek relative information. People from urban areas were more likely to seek health information. The rural people may be limited by some conditions, such as access to medical institutes, financial status, and geographical isolation (Hiebert et al., 2018). Thus, the behavioral consequences of the TMs test may be different among people with different backgrounds. These differences should be considered when applying the TMs test.

### Implications

Our study highlights the attitudes of people to the TMs test in AHEs and its effects on people, including health-behavior changes and psychological impacts. Many Chinese people receive the TMs test as part of cancer screening regardless of their needs, which is somewhat blinded. The overuse of the TMs test may bring some unexpected problems, such as overdiagnosis, which may be harmful to the asymptomatic people or patients at low risk of cancer (Mortaji and Lebduska, 2020). After getting abnormal results, even if only slightly elevated, people may develop psychological distress and intend to do more tests or even invasive exams to confirm the diagnosis, which may lead to further health care service and additional medical expenditure.

Health care providers play an important part in this scenario. People’s cancer screening behaviors could be affected by communication with health care providers (Fox et al., 2009). With the advice of doctors, people can make informed choices before receiving screening tests. Therefore, doctors should carefully identify the people who can benefit from the TMs test and provide adequate indications.

As “gate keepers” of clinical tests and examinations, doctors are expected to do the following. Before the test, doctors should collect detailed information about people’s history, symptoms, family history of cancer, and so on, to figure out whether they match the indication of the TMs test. Then, doctors should communicate appropriately with people, which includes the explanation of the function, significance, and meaning of common results to reach a joint, informed decision about whether to undergo the test. After the test, doctors should pay attention to the psychological impact on people and provide several suggestions about the next steps. Doctors should also provide information about cancer screening and health behaviors for people.

### Limitations

This study has some limitations. First, our study was conducted among people who attended AHE in the Sir Run Run Shaw Hospital, Hangzhou, Zhejiang, which is a relatively developed city in China. Given the differences in wealth and health care among various cities, our sample may not represent the situation of the whole country. Multi-centered investigations in several representative cities may be necessary to address this problem in the future.

Second, our questionnaire was distributed online, thus, the ability to use cellphones and the degree of literacy were critical to the results. There may be a potential bias in the response rate of different age groups or education levels.

Third, when the participants answered questions of the third part (behaviors about elevated TMs) of questionnaire, participants could not answer the questions exactly what they did or thought at that moment of accepting the TMs test, which leaded to the recall bias. In addition, participants might not answer the questions truthfully, which could not be recognized or avoided though online questionnaire surveys.

## Conclusion

The use of TMs test in AHEs is widely accepted by Chinese people. The TMs test may lead to positive behavior consequences (e.g., lifestyle changes, more attention on health), negative behavior consequences (e.g., unnecessary medical tests and more medical expenditure) and psychological distress. Doctors play a significant role on in this situation. Accurate indications and adequate communication with patients before and after the TMs test are in great importance to avoid negative results and psychological impacts.

## Data availability statement

The original contributions presented in the study are included in the article/Supplementary material, further inquiries can be directed to the corresponding author.

## Ethics statement

The studies involving humans were approved by the Sir Run Run Shaw Hospital Ethics Committee. The studies were conducted in accordance with the local legislation and institutional requirements. The participants provided their written informed consent to participate in this study. The animal study was approved by the Ethics Committee of Sir Run Run Shaw hospital, Zhejiang University school of Medicine. The study was conducted in accordance with the local legislation and institutional requirements.

## Author contributions

RY: Conceptualization, Data curation, Formal analysis, Investigation, Methodology, Writing – original draft, Writing – review & editing. ZX: Conceptualization, Supervision, Writing – review & editing. YL: Software, Writing – original draft. YZ: Formal analysis, Investigation, Writing – original draft. LC: Resources, Supervision, Writing – review & editing.

## Glossary

TMs: Tumor markers
AHEs: Annual health examinations
PSA: Prostate-specific antigen
CA 19–9: Carbohydrate antigen 19–9
CRC: Colorectal cancer
CEA: Carcinoembryonic antigen
CA 15–3: Carbohydrate antigen 15–3
AFP: Alpha-fetoprotein
NSE: Neurone specific enolase
CI: Confidence interval
OR: Odds ratios
CT: Computed tomography
