# Supplementary material for: Attitudes and health behaviors of middle-aged and older adults with elevated tumor markers in China
Source: Front Psychol. 2024 Feb 6;15:1265648. doi: 10.3389/fpsyg.2024.1265648 (PMC10878324; doi:10.3389/fpsyg.2024.1265648)
Supplement: Supplementary file 2 [file Data_Sheet_1.docx]

Questionnaire

Dear sir or madam,

This is a questionnaire about the tumor markers(TMs) test application in the annual health examination(AHE). Your attitude and opinion about TMs test will be helpful for its better development and application. The following questions may take you about 5 minutes and please finish them with your real thoughts. The study was approved by the Sir Run Run Shaw Hospital Ethics Committee and all your information will be strictly confidential. If you agree with above, please press the “I agree” button and finish the following questionnaire. Thank you for your cooperation.

- I agree
- No, I don’t

Section one-Basic information

Name: ________________

Telephone: _________________

Education:

- High School and below
- College degree and higher

Area of residence

- Urban
- Rural

Health insurance

- Yes
- No

Section two

Please choose the answer that best meets your opinion,.

|  | Question | strongly agree | agree | disagree | strongly disagree |
| --- | --- | --- | --- | --- | --- |
| 1 | If my doctor say there is no necessary, I would be willing to stop tumor markers test. |  |  |  |  |
| 2 | I would like to reduce the number of tumor markers in my health checkups. |  |  |  |  |
| 3 | I have some idea about the tumor markers I received. |  |  |  |  |
| 4 | I believe all of my tumor markers tests are necessary. |  |  |  |  |
| 5 | I don’t want to reduce the number of tumor markers in my health checkups. |  |  |  |  |
| 6 | I think I receive too many tumor markers tests in my health checkups. |  |  |  |  |
| 7 | I feel stressed about the result of tumor markers. |  |  |  |  |
| 8 | I believe I would like to accept tumor markers test as routine in my health checkups, and will accept even more. |  |  |  |  |

Section three

Please choose the answer that best meets your reality.

1. How was the results of TMs retest?

A. decreased or recovered

B. almost no change

C. elevated

2. Did you try changing lifestyle to keep health after the TMs test, such as choosing a healthy diet, smoking cessation, reducing alcohol consumption and more physical activities?

A. yes

B. no

3. After received the TMs test, did you ever seek health information about tumor markers?

A. yes

B. no

If the answer of question 3 is “yes”, please answer question 4, and if not, please skip to question 5.

4. what was your first approach to seek health information about tumor markers?

A. consulting doctors in the general hospitals

B. consulting family doctors

C. consulting friends

D. searching the Internet

5. If you get the elevated result of TMs test, do you want to get a retest?

A. yes

B. no

6. If you get the elevated result of TMs test, do you want to get more tests intended to confirm a diagnosis, such as enhanced CT scan, endoscopic procedures and biopsy?

A. yes

B. no
